# 6,8-Di­iodo-4-oxo-4*H*-chromene-3-carbalde­hyde

**DOI:** 10.1107/S1600536814006904

**Published:** 2014-04-12

**Authors:** Yoshinobu Ishikawa

**Affiliations:** a School of Pharmaceutical Sciences, University of Shizuoka, 52-1 Yada, Suruga-ku, Shizuoka 422-8526, Japan

## Abstract

The title compound, C_10_H_4_I_2_O_3_, is an iodinated 3-formyl­chromone derivative, and the atoms are essentially coplanar [r.m.s. deviation = 0.049 Å, largest deviation from the least-squares plane = 0.111 (9) Å for the CH(=O) C atom]. In the crystal, mol­ecules are linked into a three-dimensional network through halogen bonds [I⋯O = 3.352 (5) and 3.405 (7) Å, C—I⋯O = 144.2 (3) and 154.5 (3)°, and C=O⋯I = 134.9 (6) and 146.0 (6)°], and π–π stacking inter­actions [centroid–centroid distance = 3.527 (6) Å].

## Related literature   

For the preparation of the precursor of the title compound, see: Khansole *et al.* (2008 ▶). For related structures, see: Ishikawa & Motohashi (2013 ▶); Ishikawa (2014 ▶). For halogen bonding, see: Auffinger *et al.* (2004 ▶); Metrangolo *et al.* (2005 ▶); Wilcken *et al.* (2013) ▶; Sirimulla *et al.* (2013 ▶).
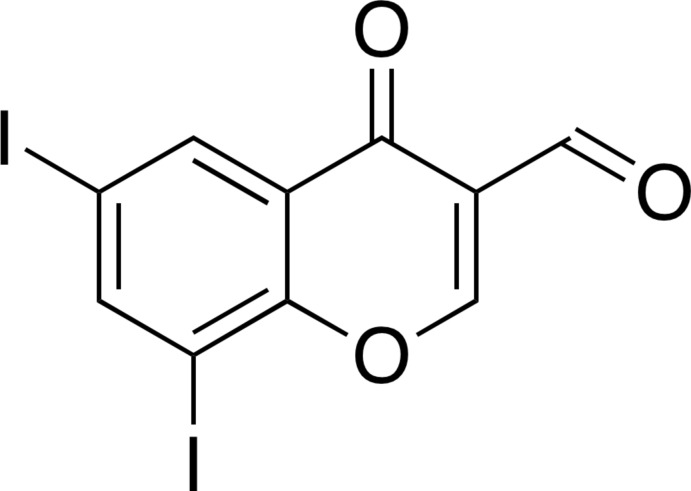


## Experimental   

### 

#### Crystal data  

C_10_H_4_I_2_O_3_
*M* *_r_* = 425.95
Triclinic, 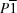

*a* = 7.290 (3) Å
*b* = 8.779 (5) Å
*c* = 9.767 (4) Å
α = 63.82 (3)°
β = 75.44 (3)°
γ = 68.05 (4)°
*V* = 517.5 (5) Å^3^
*Z* = 2
Mo *K*α radiation
μ = 6.06 mm^−1^
*T* = 100 K
0.48 × 0.30 × 0.25 mm

#### Data collection  

Rigaku AFC-7R diffractometer
Absorption correction: ψ scan (North *et al.*, 1968 ▶) *T*_min_ = 0.152, *T*_max_ = 0.220
2933 measured reflections
2383 independent reflections
2361 reflections with *F* ^2^ > 2σ(*F* ^2^)
*R*_int_ = 0.026
3 standard reflections every 150 reflections intensity decay: −1.1%

#### Refinement  

*R*[*F* ^2^ > 2σ(*F* ^2^)] = 0.051
*wR*(*F* ^2^) = 0.147
*S* = 1.23
2383 reflections
137 parameters
H-atom parameters constrained
Δρ_max_ = 3.56 e Å^−3^
Δρ_min_ = −3.01 e Å^−3^

### 

Data collection: *WinAFC Diffractometer Control Software* (Rigaku, 1999 ▶); cell refinement: *WinAFC Diffractometer Control Software*; data reduction: *WinAFC Diffractometer Control Software*; program(s) used to solve structure: *SIR92* (Altomare *et al.*, 1994 ▶); program(s) used to refine structure: *SHELXL97* (Sheldrick, 2008 ▶); molecular graphics: *CrystalStructure* (Rigaku, 2010 ▶); software used to prepare material for publication: *CrystalStructure*.

## Supplementary Material

Crystal structure: contains datablock(s) General, I. DOI: 10.1107/S1600536814006904/zl2583sup1.cif

Structure factors: contains datablock(s) I. DOI: 10.1107/S1600536814006904/zl2583Isup2.hkl

Supporting information file. DOI: 10.1107/S1600536814006904/zl2583Isup3.cml

CCDC reference: 994113

Additional supporting information:  crystallographic information; 3D view; checkCIF report

## Figures and Tables

**Fig. 1.**
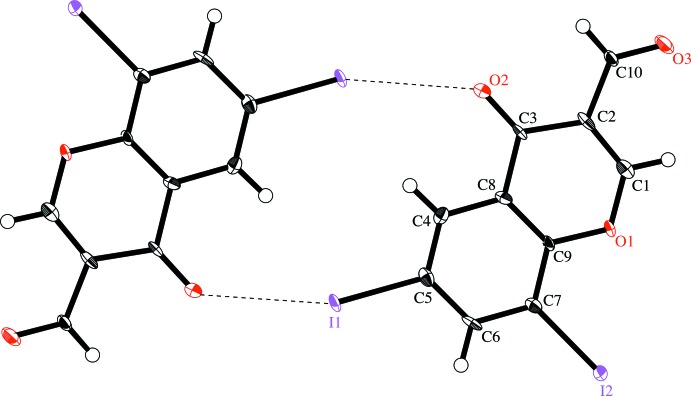
The molecular structure of the title compound, with displacement ellipsoids drawn at the 50% probability level. Hydrogen atoms are shown as small spheres of arbitrary radius. The intermolecular interaction of the title compound is represented as dashed lines for I···O.

**Fig. 2.**
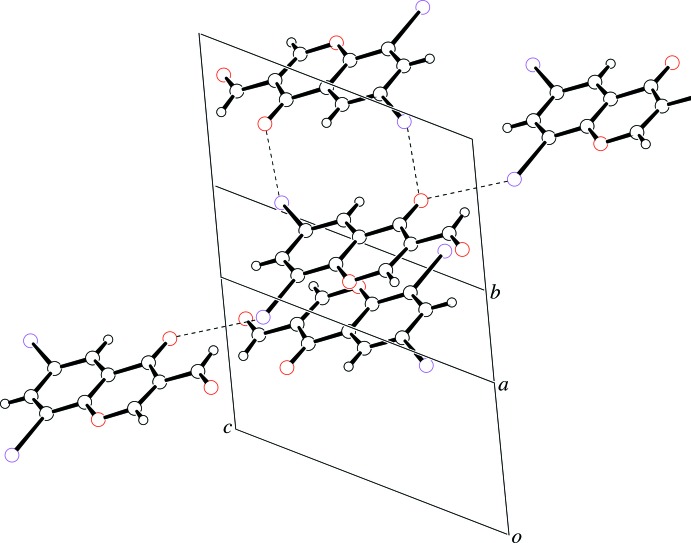
A view of the intermolecular interactions of the title compound, represented as dashed lines for I···O interactions.

## supplementary crystallographic information

### 1. Comment

Halogen bonds have been found to occur in organic, inorganic, and biological systems, and have recently attracted much attention in medicinal chemistry, chemical biology and supramolecular chemistry (Auffinger *et al.*, 2004, Metrangolo *et al.*, 2005, Wilcken *et al.*, 2013, Sirimulla *et al.*, 2013). We have recently reported the crystal structures of halogenated 3-formylchromone derivatives, 6,8-dichloro-4-oxochromene-3-carbaldehyde and 6,8-dibromo-4-oxochromene-3-carbaldehyde (Ishikawa & Motohashi, 2013; Ishikawa, 2014). It was found that these molecules are linked through halogen bonds in a similar fashion in the crystals. As part of our interest in chemical bonding, we herein report the crystal structure of 6,8-diiodo-4-oxochromene-3-carbaldehyde, which was prepared by the Vilsmeier–Haack reaction of 2-hydroxy-3,5-diiodoacetophenone with *N*,*N*-dimethylformamide (DMF) in the presence of POCl_3_ in good yield.

The mean deviation of the least-square planes for the non-hydrogen atoms is 0.0487 Å, and the largest deviations is 0.111 (9) Å for C10. These mean that the atoms are essentially coplanar.

In the crystal, the molecule is assembled through characteristic intermolecular interactions between the I1 atom at the 6-position and the O2 atom of the α,β-unsaturated carbonyl group of its inversion-symmetry equivalent [I1···O2; 3.405 (7) Å, C5–I1···O2^i^ = 154.5 (3)°, I1···O2^i^–C3^i^ = 134.9 (6)° (i): -*x* + 1, -*y* + 2, -*z* + 1, Fig. 1], and between the I2 atom at the 6-position and the O2 atom of the α,β-unsaturated carbonyl group of its translation-symmetry equivalent [I2···O2; 3.352 (5) Å, C7–I2···O2^ii^ = 144.2 (3)°, I2···O2^ii^–C3^ii^ = 146.0 (6)° (i): *x*, *y* - 1, *z+*1, Fig. 2]. The short contact and the geometry of the I···O interactions come within the range of halogen bonding (Auffinger *et al.*, 2004). It is noted that the geometry of the I···O interactions for the title compound is different from that for 6,8-dichloro-4-oxochromene-3-carbaldehyde and 6,8-dibromo-4-oxochromene-3-carbaldehyde. The three-dimensional network *via* the halogen bonds in the crystal of the title compound is more extensive. This is probably due to the larger size of the positive σ-holes of the I1 and I2 atoms (Auffinger *et al.*, 2004, Sirimulla *et al.*, 2013). The intermolecular π-π stacking interaction of the benzene ring of the molecule with that of the inversion-symmetry equivalent^iii^ is also observed [centroid–centroid distance = 3.527 (6) Å (iii): -*x* + 1, -*y* + 1, -*z* + 1], as shown in Fig. 2.

### 2. Experimental

2-Hydroxy-3,5-diiodoacetophenone was prepared according to the literature method (Khansole *et al.*, 2008). To a solution of 2-hydroxy-3,5-diiodoacetophenone (134 mmol) in DMF (5 ml) was added dropwise POCl_3_ (335 mmol) for 5 min at 0 °C. After the mixture was stirred for 15 h at room temperature, water (20 ml) was added. The precipitates were collected, washed with water and dried *in vacuo* (yield: 81.1%). ^1^H NMR (400 MHz, DMSO-*d*_6_): *δ* = 8.31 (d, 1H, *J* = 2.0 Hz), 8.63 (d, 1H, *J* = 2.0 Hz), 9.04 (s, 1H), 10.07 (s, 1H). DART-MS calcd for [C_10_H_4_I_2_O_3_ + H^+^]: 426.825, found 426.869. Single crystals suitable for X-ray diffraction were obtained by slow evaporation of an ethyl acetate solution of the title compound at room temperature.

### 3. Refinement

The C(*sp*^2^)-bound hydrogen atoms were placed in geometrical positions [C–H 0.95 Å, *U*_iso_(H) = 1.2*U*_eq_(C)], and refined using a riding model. There are large positive and negative electron densities around the iodine atoms in spite of the good *R* value. The reflection data were collected separately with a smaller sized crystal, but it is found that the large residual electron densities around the iodine atoms still remained. For most of the disagreeable reflections in the *SHELX*.lst file, *F*_obs_ is much greater than *F*_calc_. This suggests the possibility of non-merohedral twinning. Thus, the large residual electron densities could be derived from non-merohedral twinning. Unfortunately, it is difficult to confirm the possibility on a single point detector diffractometer, Rigaku AFC7*R*. One reflection (–2 7 3) was omitted because of systematic error. Extinction correction was applied for improvement of large negative electron densities and the *R* value.

### Crystal data

**Table:**

C_10_H_4_I_2_O_3_	*Z* = 2
*M**_r_* = 425.95	*F*(000) = 388.00
Triclinic, *P*1	*D*_x_ = 2.733 Mg m^−^^3^
Hall symbol: -P 1	Mo *K*α radiation, λ = 0.71069 Å
*a* = 7.290 (3) Å	Cell parameters from 25 reflections
*b* = 8.779 (5) Å	θ = 15.2–17.1°
*c* = 9.767 (4) Å	µ = 6.06 mm^−^^1^
α = 63.82 (3)°	*T* = 100 K
β = 75.44 (3)°	Block, yellow
γ = 68.05 (4)°	0.48 × 0.30 × 0.25 mm
*V* = 517.5 (5) Å^3^	

### Data collection

**Table:**

Rigaku AFC-7R diffractometer	*R*_int_ = 0.026
ω–2θ scans	θ_max_ = 27.5°
Absorption correction: ψ scan (North *et al.*, 1968)	*h* = −5→9
*T*_min_ = 0.152, *T*_max_ = 0.220	*k* = −10→11
2933 measured reflections	*l* = −12→12
2383 independent reflections	3 standard reflections every 150 reflections
2361 reflections with *F*^2^ > 2σ(*F*^2^)	intensity decay: −1.1%

### Refinement

**Table:**

Refinement on *F*^2^	Hydrogen site location: inferred from neighbouring sites
*R*[*F*^2^ > 2σ(*F*^2^)] = 0.051	H-atom parameters constrained
*wR*(*F*^2^) = 0.147	*w* = 1/[σ^2^(*F*_o_^2^) + (0.0884*P*)^2^ + 4.3985*P*] where *P* = (*F*_o_^2^ + 2*F*_c_^2^)/3
*S* = 1.23	(Δ/σ)_max_ = 0.001
2383 reflections	Δρ_max_ = 3.56 e Å^−^^3^
137 parameters	Δρ_min_ = −3.01 e Å^−^^3^
0 restraints	Extinction correction: *SHELXL97* (Sheldrick, 2008)
Primary atom site location: structure-invariant direct methods	Extinction coefficient: 0.018 (3)
Secondary atom site location: difference Fourier map	

### Special details

**Table:**

Refinement. Refinement was performed using all reflections. The weighted *R*-factor (*wR*) and goodness of fit (*S*) are based on *F*^2^. *R*-factor (gt) are based on *F*. The threshold expression of *F*^2^ > 2.0 σ(*F*^2^) is used only for calculating *R*-factor (gt).

### Fractional atomic coordinates and isotropic or equivalent isotropic displacement parameters (Å^2^)

**Table:**

	*x*	*y*	*z*	*U*_iso_*/*U*_eq_	
I1	0.32790 (6)	0.83369 (5)	0.74688 (5)	0.0152 (3)	
I2	0.74091 (6)	0.06022 (5)	0.85492 (5)	0.0179 (3)	
O1	0.9121 (7)	0.2469 (6)	0.5147 (6)	0.0131 (9)	
O2	0.8156 (8)	0.7689 (7)	0.2159 (6)	0.0180 (10)	
O3	1.2172 (8)	0.3795 (8)	0.0749 (6)	0.0197 (10)	
C1	1.0076 (10)	0.2949 (10)	0.3730 (8)	0.0163 (13)	
C2	0.9844 (9)	0.4629 (10)	0.2687 (8)	0.0129 (12)	
C3	0.8432 (9)	0.6141 (9)	0.3051 (7)	0.0109 (12)	
C4	0.6053 (10)	0.6899 (9)	0.5166 (8)	0.0125 (12)	
C5	0.5157 (10)	0.6387 (9)	0.6645 (8)	0.0135 (12)	
C6	0.5540 (10)	0.4571 (10)	0.7630 (8)	0.0146 (12)	
C7	0.6876 (10)	0.3271 (9)	0.7098 (7)	0.0128 (12)	
C8	0.7425 (9)	0.5587 (9)	0.4625 (7)	0.0118 (12)	
C9	0.7809 (9)	0.3800 (8)	0.5608 (7)	0.0104 (11)	
C10	1.1073 (10)	0.4944 (9)	0.1196 (8)	0.0128 (12)	
H1	1.1002	0.2020	0.3435	0.0196*	
H2	0.5756	0.8124	0.4509	0.0150*	
H3	0.4896	0.4234	0.8646	0.0176*	
H4	1.0995	0.6144	0.0536	0.0153*	

### Atomic displacement parameters (Å^2^)

**Table:**

	*U*^11^	*U*^22^	*U*^33^	*U*^12^	*U*^13^	*U*^23^
I1	0.0159 (3)	0.0127 (3)	0.0163 (3)	−0.00142 (18)	0.00485 (17)	−0.0107 (2)
I2	0.0180 (3)	0.0106 (3)	0.0169 (3)	−0.00243 (19)	0.00704 (18)	−0.0045 (2)
O1	0.014 (2)	0.010 (2)	0.013 (2)	−0.0006 (17)	0.0030 (17)	−0.0082 (17)
O2	0.019 (3)	0.016 (3)	0.012 (3)	−0.0030 (19)	0.0043 (18)	−0.0046 (19)
O3	0.017 (3)	0.026 (3)	0.015 (3)	−0.003 (2)	0.0040 (18)	−0.012 (2)
C1	0.014 (3)	0.021 (4)	0.016 (3)	−0.004 (3)	0.001 (3)	−0.012 (3)
C2	0.007 (3)	0.023 (4)	0.015 (3)	−0.006 (3)	0.004 (3)	−0.015 (3)
C3	0.008 (3)	0.016 (3)	0.010 (3)	−0.003 (3)	0.003 (2)	−0.010 (3)
C4	0.015 (3)	0.009 (3)	0.013 (3)	−0.002 (3)	−0.005 (3)	−0.004 (3)
C5	0.011 (3)	0.016 (3)	0.018 (3)	−0.005 (3)	0.002 (3)	−0.011 (3)
C6	0.014 (3)	0.026 (4)	0.010 (3)	−0.010 (3)	0.006 (2)	−0.012 (3)
C7	0.014 (3)	0.016 (3)	0.011 (3)	−0.006 (3)	0.002 (3)	−0.007 (3)
C8	0.013 (3)	0.015 (3)	0.009 (3)	−0.007 (3)	0.003 (3)	−0.006 (3)
C9	0.013 (3)	0.010 (3)	0.011 (3)	−0.003 (3)	0.002 (3)	−0.008 (3)
C10	0.012 (3)	0.015 (3)	0.010 (3)	−0.001 (3)	0.001 (3)	−0.008 (3)

### Geometric parameters (Å, º)

**Table:**

I1—C5	2.088 (8)	C4—C5	1.373 (9)
I2—C7	2.077 (7)	C4—C8	1.420 (10)
O1—C1	1.340 (8)	C5—C6	1.412 (9)
O1—C9	1.377 (9)	C6—C7	1.399 (11)
O2—C3	1.219 (8)	C7—C9	1.392 (9)
O3—C10	1.208 (10)	C8—C9	1.392 (8)
C1—C2	1.348 (9)	C1—H1	0.950
C2—C3	1.470 (10)	C4—H2	0.950
C2—C10	1.474 (9)	C6—H3	0.950
C3—C8	1.476 (9)	C10—H4	0.950
			
I2···O1	3.150 (5)	C9···C5^v^	3.539 (13)
O1···C3	2.884 (8)	C10···O3^vii^	2.980 (10)
O2···C1	3.566 (9)	C10···C5^ii^	3.421 (11)
O2···C4	2.862 (8)	C10···C6^ii^	3.199 (13)
O2···C10	2.895 (9)	C10···C7^ii^	3.574 (14)
O3···C1	2.840 (8)	C10···C10^vii^	3.072 (13)
C1···C7	3.579 (10)	I1···H2	3.0418
C1···C8	2.730 (11)	I1···H3	3.0886
C2···C9	2.778 (9)	I2···H3	3.0562
C4···C7	2.811 (9)	O2···H2	2.5939
C5···C9	2.772 (10)	O2···H4	2.6052
C6···C8	2.806 (9)	O3···H1	2.5096
I1···O2^i^	3.405 (7)	C1···H4	3.2682
I1···O3^ii^	3.594 (6)	C3···H1	3.2906
I2···O2^iii^	3.352 (5)	C3···H2	2.6671
I2···O3^iv^	3.514 (7)	C3···H4	2.6866
O1···C3^ii^	3.587 (12)	C4···H3	3.2790
O1···C5^v^	3.557 (10)	C6···H2	3.2839
O2···I1^i^	3.405 (7)	C9···H1	3.1762
O2···I2^vi^	3.352 (5)	C9···H2	3.2812
O2···C7^ii^	3.585 (10)	C9···H3	3.2682
O2···C9^ii^	3.599 (10)	C10···H1	2.5423
O3···I1^ii^	3.594 (6)	H1···H4	3.4744
O3···I2^iv^	3.514 (7)	I1···H2^i^	3.0877
O3···O3^vii^	3.353 (8)	I1···H4^ix^	3.2019
O3···C2^vii^	3.493 (10)	I2···H1^iv^	3.3483
O3···C5^ii^	3.492 (12)	O1···H1^iv^	3.5832
O3···C6^viii^	3.381 (8)	O3···H3^viii^	2.4768
O3···C6^ii^	3.531 (13)	O3···H3^ii^	3.4952
O3···C10^vii^	2.980 (10)	O3···H4^vii^	2.8657
C1···C4^ii^	3.329 (13)	C1···H2^ii^	3.4833
C1···C8^ii^	3.548 (14)	C3···H3^v^	3.4417
C2···O3^vii^	3.493 (10)	C4···H1^ii^	3.3938
C2···C4^ii^	3.580 (11)	C5···H1^ii^	3.5387
C2···C5^ii^	3.571 (11)	C6···H4^ii^	3.1756
C2···C8^ii^	3.577 (12)	C7···H2^v^	3.5512
C2···C9^ii^	3.581 (14)	C7···H4^ii^	3.3859
C3···O1^ii^	3.587 (12)	C10···H3^viii^	3.3078
C3···C6^v^	3.451 (13)	C10···H3^ii^	3.3304
C3···C9^ii^	3.346 (12)	C10···H4^vii^	3.1352
C4···C1^ii^	3.329 (13)	H1···I2^iv^	3.3483
C4···C2^ii^	3.580 (11)	H1···O1^iv^	3.5832
C4···C7^v^	3.518 (13)	H1···C4^ii^	3.3938
C4···C9^v^	3.413 (12)	H1···C5^ii^	3.5387
C5···O1^v^	3.557 (10)	H1···H2^ii^	3.4018
C5···O3^ii^	3.492 (12)	H2···I1^i^	3.0877
C5···C2^ii^	3.571 (11)	H2···C1^ii^	3.4833
C5···C9^v^	3.539 (13)	H2···C7^v^	3.5512
C5···C10^ii^	3.421 (11)	H2···H1^ii^	3.4018
C6···O3^ix^	3.381 (8)	H2···H2^i^	3.5393
C6···O3^ii^	3.531 (13)	H3···O3^ix^	2.4768
C6···C3^v^	3.451 (13)	H3···O3^ii^	3.4952
C6···C8^v^	3.526 (13)	H3···C3^v^	3.4417
C6···C10^ii^	3.199 (13)	H3···C10^ix^	3.3078
C7···O2^ii^	3.585 (10)	H3···C10^ii^	3.3304
C7···C4^v^	3.518 (13)	H3···H3^x^	3.5106
C7···C8^v^	3.533 (11)	H3···H4^ix^	3.2916
C7···C10^ii^	3.574 (14)	H3···H4^ii^	3.1545
C8···C1^ii^	3.548 (14)	H4···I1^viii^	3.2019
C8···C2^ii^	3.577 (12)	H4···O3^vii^	2.8657
C8···C6^v^	3.526 (13)	H4···C6^ii^	3.1756
C8···C7^v^	3.533 (11)	H4···C7^ii^	3.3859
C9···O2^ii^	3.599 (10)	H4···C10^vii^	3.1352
C9···C2^ii^	3.581 (14)	H4···H3^viii^	3.2916
C9···C3^ii^	3.346 (12)	H4···H3^ii^	3.1545
C9···C4^v^	3.413 (12)	H4···H4^vii^	3.4680
			
C1—O1—C9	117.8 (5)	C3—C8—C4	119.6 (6)
O1—C1—C2	126.0 (7)	C3—C8—C9	121.5 (6)
C1—C2—C3	120.3 (6)	C4—C8—C9	118.9 (6)
C1—C2—C10	119.5 (7)	O1—C9—C7	116.8 (6)
C3—C2—C10	120.2 (6)	O1—C9—C8	121.5 (6)
O2—C3—C2	123.5 (6)	C7—C9—C8	121.7 (6)
O2—C3—C8	123.7 (7)	O3—C10—C2	125.0 (7)
C2—C3—C8	112.8 (5)	O1—C1—H1	116.984
C5—C4—C8	119.7 (6)	C2—C1—H1	116.981
I1—C5—C4	119.2 (5)	C5—C4—H2	120.173
I1—C5—C6	119.6 (5)	C8—C4—H2	120.164
C4—C5—C6	121.1 (7)	C5—C6—H3	120.281
C5—C6—C7	119.4 (6)	C7—C6—H3	120.280
I2—C7—C6	119.1 (5)	O3—C10—H4	117.476
I2—C7—C9	121.7 (5)	C2—C10—H4	117.479
C6—C7—C9	119.2 (6)		
			
C1—O1—C9—C7	−178.3 (7)	C8—C4—C5—I1	176.2 (7)
C1—O1—C9—C8	2.2 (11)	C8—C4—C5—C6	−1.5 (13)
C9—O1—C1—C2	−1.6 (13)	H2—C4—C5—I1	−3.8
C9—O1—C1—H1	178.5	H2—C4—C5—C6	178.5
O1—C1—C2—C3	−0.3 (14)	H2—C4—C8—C3	1.8
O1—C1—C2—C10	177.4 (8)	H2—C4—C8—C9	−179.1
H1—C1—C2—C3	179.7	I1—C5—C6—C7	−176.9 (5)
H1—C1—C2—C10	−2.6	I1—C5—C6—H3	3.1
C1—C2—C3—O2	179.9 (8)	C4—C5—C6—C7	0.9 (13)
C1—C2—C3—C8	1.4 (12)	C4—C5—C6—H3	−179.1
C1—C2—C10—O3	7.6 (14)	C5—C6—C7—I2	−179.4 (7)
C1—C2—C10—H4	−172.4	C5—C6—C7—C9	0.4 (13)
C3—C2—C10—O3	−174.7 (8)	H3—C6—C7—I2	0.6
C3—C2—C10—H4	5.3	H3—C6—C7—C9	−179.6
C10—C2—C3—O2	2.3 (13)	I2—C7—C9—O1	−0.7 (11)
C10—C2—C3—C8	−176.3 (7)	I2—C7—C9—C8	178.8 (5)
O2—C3—C8—C4	−0.2 (13)	C6—C7—C9—O1	179.5 (7)
O2—C3—C8—C9	−179.3 (8)	C6—C7—C9—C8	−1.0 (13)
C2—C3—C8—C4	178.4 (7)	C3—C8—C9—O1	−1.1 (12)
C2—C3—C8—C9	−0.7 (11)	C3—C8—C9—C7	179.5 (7)
C5—C4—C8—C3	−178.2 (7)	C4—C8—C9—O1	179.8 (7)
C5—C4—C8—C9	0.9 (12)	C4—C8—C9—C7	0.3 (12)

Symmetry codes: (i) −*x*+1, −*y*+2, −*z*+1; (ii) −*x*+2, −*y*+1, −*z*+1; (iii) *x*, *y*−1, *z*+1; (iv) −*x*+2, −*y*, −*z*+1; (v) −*x*+1, −*y*+1, −*z*+1; (vi) *x*, *y*+1, *z*−1; (vii) −*x*+2, −*y*+1, −*z*; (viii) *x*+1, *y*, *z*−1; (ix) *x*−1, *y*, *z*+1; (x) −*x*+1, −*y*+1, −*z*+2.
